# ALS/FTD: Evolution, Aging, and Cellular Metabolic Exhaustion

**DOI:** 10.3389/fneur.2022.890203

**Published:** 2022-05-27

**Authors:** Robert David Henderson, Kasper Planeta Kepp, Andrew Eisen

**Affiliations:** ^1^Department of Neurology, Royal Brisbane and Women's Hospital, Brisbane, QLD, Australia; ^2^Department of Chemistry, Technical University of Denmark, Kongens Lyngby, Denmark; ^3^Division of Neurology, Department of Medicine, Faculty of Medicine, University of British Columbia, Vancouver, BC, Canada

**Keywords:** motor neuron disease, amyotrophic lateral sclerosis, evolution, protein, metabolism, aging

## Abstract

Amyotrophic lateral sclerosis and frontotemporal dementia (ALS/FTD) are neurodegenerations with evolutionary underpinnings, expansive clinical presentations, and multiple genetic risk factors involving a complex network of pathways. This perspective considers the complex cellular pathology of aging motoneuronal and frontal/prefrontal cortical networks in the context of evolutionary, clinical, and biochemical features of the disease. We emphasize the importance of evolution in the development of the higher cortical function, within the influence of increasing lifespan. Particularly, the role of aging on the metabolic competence of delicately optimized neurons, age-related increased proteostatic costs, and specific genetic risk factors that gradually reduce the energy available for neuronal function leading to neuronal failure and disease.

## Introduction

Finely controlled fractionated muscle movement enables humans to perform complex activities that require precise voluntary execution of force and speed of movement (1). The anatomical basis for this is the corticomotoneuronal system, which in humans connects monosynaptically with all motor neuron pools except those innervating the ocular and sphincter muscles (2, 3).

Both advanced cognition and a versatile motor repertoire were critical to the success of human evolution, which involved a rapid expansion of cerebral network connectivity occurring within the constraints of a bony cranium (4). The relatively rapidly evolving brain incurred increased metabolic demands (5), and selection pressures relating to human migration within and out of Africa.

Since the mid-nineteenth century, recognition of devastating diseases, including amyotrophic lateral sclerosis (ALS) and frontotemporal dementia (FTD), have emerged. These predominantly involve frontal and prefrontal neurons, but their pathology extends beyond these regions. Clinical, genetical, and biochemical features of these diseases converge on protein misfolding and metabolic dysfunction as common end points associated with impaired neuronal dysfunction (6).

This article briefly (1) outlines the evolution of the frontal and prefrontal cortex with respect to communication and language, and motor function specific to humans; (2) considers the roles played by evolutionary changes, cerebral metabolism, and senescence in an era of increasing lifespan; (3) raises the concept of possible neuron exhaustion, with proteomic cost minimization as a selective force challenged with increasing age; and (4) links this idea to ALS and FTD.

We hypothesize that these disorders and other neurodegenerations reflect in part a mismatch between evolved neocortical cellular and metabolic processes at a protein level, in the context of rapid and ever-increasing complexity of human interaction, and the relatively recent increased human lifespan. For conciseness, we do not attempt to detail genetic components of these diseases (7), nor do we consider their overlap with other neurodegenerative diseases (8).

A unified etiology of ALS/FTD is proposed that implicates evolutionary optimized neurons, metabolically challenged by RNA/protein turnover in certain risk phenotypes leading to neuronal exhaustion and disease. We suggest that the hypothesis can be expanded with more data and point toward metabolism and protein turnover as potentially key targets for efficient treatment paradigms.

## Evolution of Cognitive and Motor Function

The evolution of cognitive function and brain development has resulted from the complex interplay of nature and nurture, where development seems to be driven by genes and shaped by the environment (9, 10). Modern humans have enhanced cognitive functioning, especially in the domains of cooperation, theory of mind, language, and culture (11), and are capable of processing vast information and solving abstract problems (12–14). Higher order cognitive skills of humans evolved through the separation of humans from earlier hominid lineages (15). Whether this was through adaptations of existing systems or the creation of new ones is undetermined (12, 16).

Language probably evolved out of gesture as a protolanguage (17–20). Gesture is universal to the animal kingdom. Some gestures are individual, but many are common to a specific language and others are common to all humans. Newborns and infants largely communicate with gestures accompanied by non-verbal vocalization, and children learn language through social interaction and gain practice using sentence constructions that have been created by linguistic communities over time.

Complex forms of communication, especially human language, defines one of the most difficult problems for evolutionary biology (21–23). Language is a particularly remarkable outcome of the evolution of cognitive complexity and requires perceiving the external world in terms of objects and actions and naming them using a set of signals. Even though human communication (gestures and language) is far more structured and complex than seen in other animals, there are no specific physiological, neurological, or genetic traits that explain the human communication, executive functioning, and abstract thinking skills that have evolved during the latest 100,000 years (12). But it has been suggested that speech could be more cost-effective compared to gesture and developed progressively as group size increased (24).

The basic layout of the larynx and vocal tract is highly conserved and virtually homologous in both form and function among all terrestrial mammals, including humans (25). Indeed, the macaque vocal apparatus is “speech ready,” capable of producing an adequate range of speech sounds to support spoken language (26). However, only humans have developed voluntary control of the larynx (27, 28). This required the unique expansion of fast-conducting monosynaptic corticomotoneuronal connections which in humans occurs for all motor neuron pools, except those of the external ocular muscles and bladder wall (29).

The greatly expanded corticomotoneuronal system with associated neo-cortical networks underlies finely tuned motor control of hand function (and thus use tool, play musical instruments, and paint), the ability to traverse uneven terrain, and for example, to skate, ski, and play professional football, and employ diversified vocalization enabling variable pitch, tone, velocity of speech and loudness, in a complex association with respiratory function (29, 30).

Vocal cues are a rich source of information about a speaker's emotional state. The term “prosody” refers to the changes in pitch, loudness, rhythm, and voice quality corresponding to a person's emotional state (31–33). The relationship between vocal complexity and brain architecture across non-human primates also has relevance to the evolution of human speech. A positive correlation has been recently observed between vocal repertoire scope and the relative size of cortical association areas which governs voluntary control (28).

The motor and premotor areas of the human and non-human primate cortex are engaged not only in preparation and execution of voluntary movement but also perform fundamental computations associated with executive function and other cognitive aspects of behavior (34). Furthermore, the incremental diversification of motor areas in humans is accompanied by the emergence of new cognitive abilities. In particular, primate motor regions not only control the low-level aspects of planning and control of movements but also participate in the perceptual and motor aspects of sophisticated cognitive functions such as decision-making, action understanding/imitation, and language (10, 35–37).

Humans have evolved a finely tuned pincer grip, utilizing the thumb and index finger. Impairment of this can be an early, unique feature of ALS, referred to as the split hand (29). Similarly, early loss of foot dorsiflexion and elbow flexion, referred to as split foot and elbow, may also be early impairments in ALS (38). The motor units subserving these movements have the strongest corticomotoneuronal drive, and it has been proposed that in ALS, there is loss of muscle synergies subserved by motor units with the strongest corticomotoneuronal drive (3).

It has been hypothesized that the evolution of cognition increased the returns from cooperating to the point where the benefits to self were sufficient for cooperation to remain stable as the group size increased and the relatedness decreased (39), with the higher cognitive needs of expanded, non-kin cooperation developing slowly. This change to co-operative behavior could be accomplished with more versatile communication (40).

Through evolution, hominin brain sizes smaller than homo sapiens remained stable at 400–500 cc until about 2 million years ago (41). The human brain size reaches its adult dimensions by 3–4 years, similar to chimpanzees yet human frontal lobe development continues until at least the early 20s (42–45). At a macro level, as human species developed improved frontal lobe function, including theory of mind, any change in cranial size was comparatively minor (46) and roughly scaled to the body size of primates (47). Within this fixed cranial capacity, cortical size could be increased by folding (48) while also allowing shorter axonal distances for the rapid connectivity needed in the frontal lobes.

At a micro level, the functional capacity of a neuronal structure is inherently limited by its neural architecture and signal processing time (48). An important component of the frontal lobe development was the discrete modifications in local circuitry and interconnectivity of selected parts of the brain which became highly organized in humans (44). The scaling of the number and distribution of neurons is an important component through evolution (41), with a greater scaling of the number of cortical motor neurons through primate evolution (49).

## Environmental Factors at a Macro Level—Evolution, Aging, and Energy Metabolism of Motor Neurons

As modern humans migrated out of the African sub-continent into the colder habitats, there was pressure to modify cerebral energy metabolism in a brain that was progressively increasing its metabolic demands in comparison to other body organs (50). Within the constraints on cranium size, most changes in cognitive function were probably associated with altered neuronal networks (5). However, neurons with many synaptic connections and high-synaptic activity are very energy-demanding, and thus, vulnerable to energy-deficiency induced by genetic and environmental risk factors (51, 52).

Complex variations in the dietary intake associated with the habitat's wildlife and foraging options and diverse cultural and technological impacts (e.g., cooking) contribute to this evolutionary history (5, 53, 54). Also, metabolic adaptations in response to human migration to colder environments may have occurred more recently. For example, in the Scandinavia human settlement occurred perhaps 5,000–10,000 years ago as the polar ice cap receded (55).

In contrast to human evolution occurring over tens of thousands of years, the recent reduced mortality and associated increased longevity has been rapid, experienced predominantly by the last four generations of humans that have ever lived (56, 57). Progress in lowering human mortality is on par with or exceeds that made in the laboratory *via* various selection and dietary restriction experiments and endocrine pathway mutations (56). The change has been largely achieved by removing environmental challenges, making injuries, and illnesses less fatal, by improving nutrition and reducing disease at younger ages and also enhancing health in the elderly (58). As a result, there has been a considerable increase in humans reaching senescent ages with vulnerability to neurodegenerative diseases (57).

Intrinsic to aging is a slowing of cerebral metabolism (8, 59–62). Recent findings suggest that disruption of neuronal homeostasis, mainly due to deficient energy metabolism, underlies neurodegeneration (63–65). Although senescent neurons may remain metabolically active and continue to function within the neuronal network, their reduced metabolic efficiency will plausibly impact overall network integrity and ultimately cognitive performance. In addition, the senescent neurons excrete a plethora of molecules that affect the function of nearby cells and provoke local inflammation potentiating the destruction of the human brain networks (65).

## The Molecular Level—Proteostasis of Neuronal Networks

Following DNA transcription, RNA molecules within a cell are bound by distinct sets of RNA-binding proteins that have the task of regulating the correct processing, transport, stability, and function/translation of proteins up to its final degradation. Proteins reach a native state but can change their folded structure if the environment changes (protein misfolding) leading to aggregates (66, 67). Mutations can also induce conformational changes and aggregation (68).

These cellular processes that maintain normal neuronal physiology throughout life are diverse, and exponentially fail with increasing age (69). Increasing age is associated with accumulation of protein aggregates characteristic of neurodegenerations but with different protein signaling pathways affected, depending upon the unfolded protein response (69, 70). Therefore, it is not surprising that the recent increase in human lifespan has been associated with increasingly prevalent cerebral protein aggregation (71).

It has been widely assumed that protein misfolding is pathogenic *via* some specific molecular toxicity as larger aggregates (i.e., protein deposits) or as more recently accepted, in the intracellular oligomeric state, with suggestions including interaction with other proteins and cell membranes (68). However, so far, the direct pathogenic species and mode of action remains obscure and therapeutic approaches that have been developed toward targeting the misfolded proteins directly, have so far met with the little clinical success (72).

Indeed, it has been proposed that the protein deposits may in some cases be beneficial as they contribute to reducing the pool of intracellular pathogenic oligomers, regardless of the mechanism of their pathogenicity (molecular toxicity or turnover costs), and outside the cells, these deposits will be less likely to interfere with cellular functions, and also less costly as they would be less targeted (and less accessible) to the intracellular proteases (73). In studies of TAR DNA binding protein 43 (TDP-43), beneficial effects of protein aggregation has also been observed (66, 68), suggesting that the toxicity occurs *via* a non-aggregated state, which we propose below is a state that is more easily subject to costly turnover.

Another recently suggested mechanism of protein-misfolding pathology employs the general metabolic cost (in ATP) of misfolded protein turnover within the cells (51). This effectively reduces energy available for basic housekeeping and cell-signaling purposes. Since the human body uses ~20% of its total energy budget on protein turnover (74), the energy costs of handling the misfolded proteins, rather than the protein's molecular toxicity *per se*—could be pathogenic. This is particularly relevant in the context of the most energy-demanding cells such as neurons, where the ATP costs of proteostasis would be first felt due to the large energy demands for inter-neuronal signaling *via* ion pumps (perhaps 50% of energy budget) (75).

The energy costs of maintaining the proteome (translation, transcription, and RNA and protein turnover) defines 20–70% of the cellular energy budget of various organisms and thus has probably been under heavy selection pressure for minimization (76). First in simple organisms to maximize energy available for cell maintenance and reproduction, and later to maximize survival of higher organisms, e.g., *via* reduced foraging needs and cognitive capacity.

Whereas, energy costs are not normally problematic, neuronal networks harbor some of the most energy-requiring cells in the human body (51, 77). It is plausible that selectively vulnerable networks of motor, or other, neurons, evolutionarily optimized for delicate metabolic competency, become challenged by lifestyle or genetic risk factors. They then may be subject to additional exhaustion caused by elevated RNA or protein turnover resulting in neuronal necrosis and network malfunction. The high-proteome turnover required to keep homeostasis in the presence of a highly abundant, repeatedly synthesized molecule *via* a repeat expansion or an unstable protein product, could be envisioned to contribute ATP costs to neurons operating near maximal capacity, and possibly accelerating aging-induced deterioration of involved networks (51).

A lack of energy could conceivably cause both depolarization of neurons, promoting excitotoxicity, a recognized event in ALS (77), but also contribute to longer-term oxidative stress and chemical imbalances that could further aggravate disease. Subsequent neuronal rewiring, which itself costs ATP as it is tightly coupled to aerobic glycolysis in energy cost-benefit tradeoffs (78), would accelerate disease progression given the metabolic deficiency cascade. Furthermore, the buildup of aggregated RNA molecules or protein copies could reflect the lack of sufficient energy available to maintain proteostasis as other energy costs increase, but also reflect the possible direct contribution of these turnover costs to the disease state. The recent studies linking proteopathy to elevated aerobic glycolysis in the human brain supports such a proteopathy-energy–cost relationship (79).

## Relevance to ALS and FTD Pathology

ALS/FTD is recognized as complex polygenic disorders (80), the genetic component perhaps contributing ~50−60% of the risk (81). The known susceptibility genes appear to have different frequencies according to race (82). The most common ALS/FTD gene, is the c9orf72 mutation which links sporadic and genetic forms of ALS and FTD (83, 84). It has a higher prevalence in the far northern European population; an evolutionary recent migration. It has been proposed that the c9orf72 expansion occurred only once in the past, with estimates varying from 1,500 to 6,000 years ago (85–87). In the USA, the median age of onset of ALS/FTD patients with the C9orf72 expansion is 58 years, an age rarely reached until after 1900 (85).

The common risk genes identified in frontal lobe pathophysiology function in molecular pathways related to RNA-metabolism and proteostasis including autophagy/proteasome, vesicle trafficking and RNA-metabolism/homeostasis (88–90). In particular, the cellular accumulation of the DNA/RNA binding protein TDP-43 found in 98% of ALS cases highlights the importance of DNA/RNA-homeostasis in the neurons (91–93).

Aging is a major risk factor for ALS/FTD and other neurodegenerations (69, 94), and there is a substantial overlap of the genetic changes in the frontal lobe diseases and the genes regulating different pathways relevant in aging. These include autophagy, inflammation, nuclear-cytoplasmic transport, and RNA processing (69, 95, 96). The recent work has identified changes in the cerebral metabolism intrinsic to both ALS and FTD (8, 59–62) with an apparent selective vulnerability of the motor neurons to energetic stress (90, 97, 98).

The central role of proteopathy and RNA homeostasis in ALS/FTD is well-indicated by the genetic risk factors (6). SOD1 mutations are a risk factor for fALS that have been shown to induce loss of protein stability and protein misfolding consistent with increased costs of managing this protein which is one of the most highly expressed in the human body with important functions in anti-oxidant stress (51). Overexpression of wild-type SOD1 contributes to the mitochondrial dysfunction and motor defects in mice due to fALS SOD1 mutations, providing support for protein abundance/turnover rather than a specific molecular toxicity of mutants being pathogenic, and bridging phenotypes of sporadic (patients harboring wild-type proteins) and fALS (patients harboring inherited additionally severe mutations).

The GGGGCC hexanucleotide repeat expansion on chromosome 9, C9orf72, is the most common genetic risk factor for fALS and is associated with abnormal protein/RNA processing (81). Since these RNA management costs would consume energy, it is plausible that they could contribute to exhaustion of the motor neurons if the hexanucleotide expansion is continuously produced and degraded (99). TDP-43, another risk factor for ALS is an important protein in transcription control known to aggregate in ALS, and thus, consistent with such a mechanism. Other genetic risk factors such as FUS/TLS and ubiquilin-2 have also been associated with proteostasis (6).

In support of the protein/RNA turnover contributing to the disease state, proteasome inhibition has been found to prevent the pathogenicity of a fALS-causing SOD1 variant, whereas removal of the inhibitor (which would reinstate protein turnover) was associated with aggravation of disease (100).

**Figure 1:**
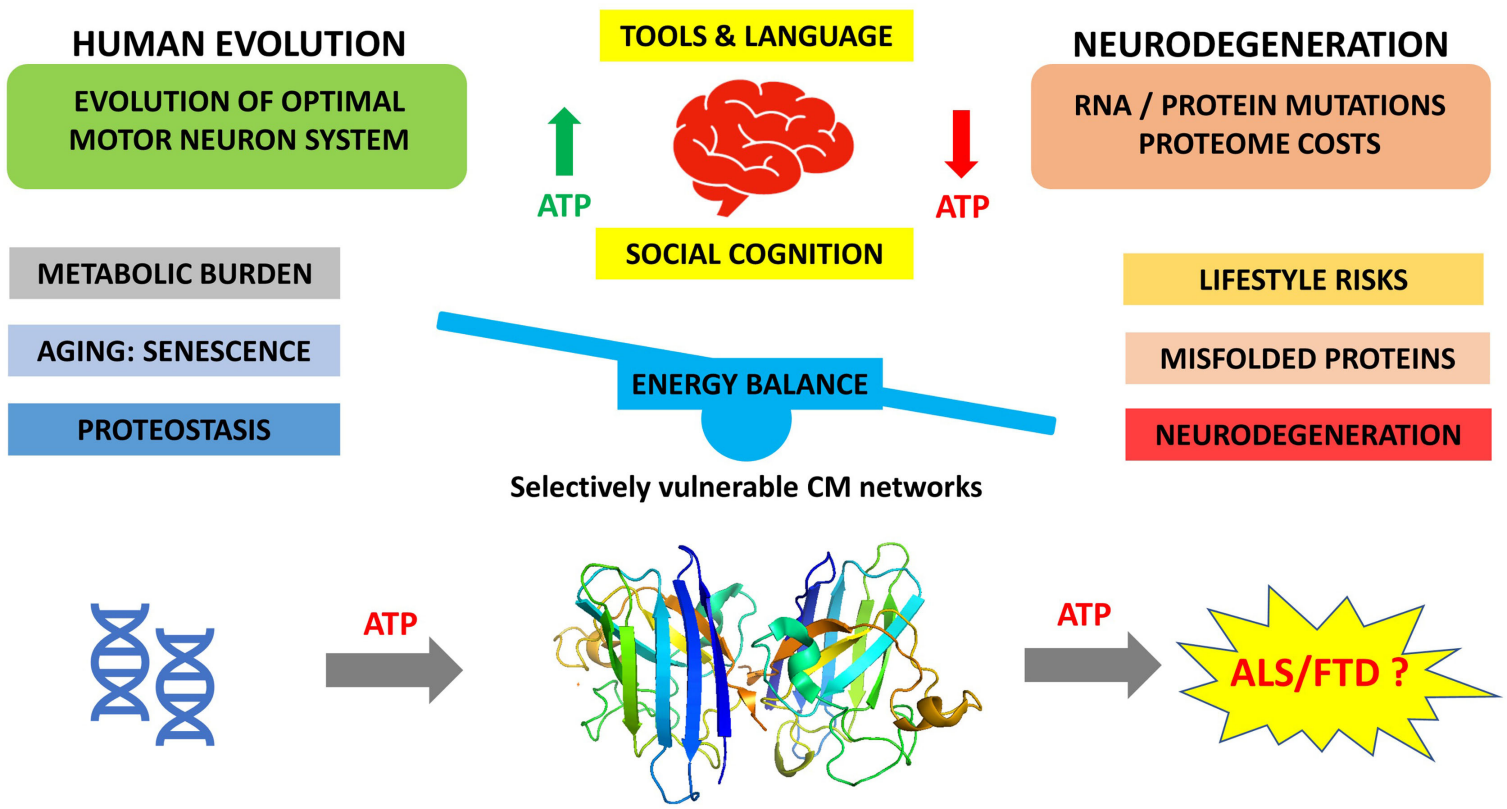
Cost-saving balance of human neural circuits resulting from evolution of human cognition and challenged by increasing age of modern humans.

At the clinical level, ALS has been associated with a hypermetabolic presentation that could suggest elevated metabolic costs during pathogenesis (62, 94, 101). It is also notable that low body mass index has been consistently identified as a risk factor for ALS, and high BMI has been associated with lower risk of ALS (102). Although it is not clear if this association is causative, these clinical features of ALS would be consistent with a hypothesis that evolutionary-optimized metabolic demands being exhausted by age-induced proteostasis costs in energy-demanding motor neurons. In our opinion, this etiology, which we have summarized in Figure 1, integrates and rationalizes both the evolutionary history of human cognition and aging, the senescence-induced proteostatic and metabolic challenges associated with this evolutionary process, and its relationship with the clinical state of ALS/FTD.

## Conclusion

Frontal and prefrontal lobe diseases are predominantly disorders of the aging nervous system. With the recent increase in longevity, largely determined by adequate shelter, good nutrition, medical advances, and reduced mortality in early life, the incidence of these neurodegenerations has increased. Extended longevity in the recent generations is unlikely to simply reflect the Darwinian natural selection, nor the Hamiltonian inclusive fitness (103). As humans age, neocortical neurons are particularly vulnerable to the effects of senescence, which include impaired energy metabolism homeostasis. This results in functional cellular failure and ultimately clinical disease. The cascade of events that determine cellular senescence are poorly defined, but among other factors, a genetic predisposition is likely relevant.

We propose that protein aggregation, the hallmark of neurodegenerations such as ALS and FTD, occurs because of the increasing metabolic burden accompanying neuronal proteostasis. This in turn is a consequence of the intersection of the evolving human brain in response to evolutionary and environmental pressures, and increasing age, which over time leads to metabolic exhaustion of energy-demanding neocortical neurons (Figure 1).

A possible mechanism for the protein aggregation lies in the energy costs of misfolded protein turnover, but other possibilities exist. While general proteasome inhibition is not a valid therapeutic strategy for these diseases, it does suggest that the burden of RNA/protein turnover could be a contributing factor in the etiology, consistent with the perspective provided earlier.

## Data Availability Statement

The raw data supporting the conclusions of this article will be made available by the authors, without undue reservation.

## Author Contributions

RH conceived the concept. KK created the Figure. All authors contributed to writing and reviewing the manuscript.

## Conflict of Interest

The authors declare that the research was conducted in the absence of any commercial or financial relationships that could be construed as a potential conflict of interest.

## Publisher's Note

All claims expressed in this article are solely those of the authors and do not necessarily represent those of their affiliated organizations, or those of the publisher, the editors and the reviewers. Any product that may be evaluated in this article, or claim that may be made by its manufacturer, is not guaranteed or endorsed by the publisher.
